# CRISPR‐driven diagnostics: Molecular mechanisms, clinical efficacy and translational challenges

**DOI:** 10.1002/ctm2.70482

**Published:** 2025-09-26

**Authors:** Zilong Wang, Qianqian Wang, Jiaming Zhang, Bingyu Li, Yuke Li, Zhengbo Chen, Dandan Guo, Shuying Feng

**Affiliations:** ^1^ Medical College Henan University of Chinese Medicine Zhengzhou China; ^2^ Henan Engineering Research Center for Chinese Medicine Foods for Special Medical Purpose Zhengzhou China

**Keywords:** bacteria, CRISPR/Cas, detect, nucleic acid test

## Abstract

**Background:**

In the realm of public health, among the primary perils menacing human well‐being, the issue of pathogen infection persists as a significant concern. Precise and timely diagnosis of diseases constitutes the bedrock for effective therapeutic interventions and epidemiological monitoring. Hence, it is crucial to develop quick, sensitive, and highly effective methods for identifying pathogen and their variants.

**Material and methods:**

This article reviews the recent research progress in the CRISPR/Cas system for detecting nucleic acids, with an emphasis on CRISPR/Cas9, CRISPR/Cas12, and CRISPR/Cas13. Initially, we provided a concise overview of the nucleic acid detection mechanism utilizing the CRISPR/Cas system. Subsequently, we dissect the molecular mechanisms of CRISPR tools, compare their clinical efficacy against traditional methods, and explore frontier innovations such as amplification‐free detection and AI integration.

**Conclusion:**

Ultimately, we argue that CRISPR diagnostics must evolve beyond technical optimization to embrace ecological adaptability, ensuring that precision medicine serves as a bridge‐rather than a barrier‐to global health equity.

**Key points:**

- **Core Mechanism**: Explains the molecular basis of CRISPR‐Cas (Cas9, Cas12, Cas13) for nucleic acid detection, leveraging crRNA‐guided targeting and trans‐cleavage activity for ultra‐sensitive (aM level) and specific pathogen identification.
- **Superior Performance**: Outperforms traditional methods in speed, sensitivity, and cost, making it ideal for point‐of‐care use in resource‐limited settings.
- **Cutting‐Edge Innovations**: Covers key advances like amplification‐free detection, portable device integration, and multiplex platforms.
- **Translation Challenges**: Discusses hurdles in clinical adoption, including inhibitor interference in complex samples, scalability limitations, the need for multi‐center clinical data, and varying regional regulations.
- **Future Outlook**: Highlights emerging directions such as integrated “sample‐to‐result” systems and AI integration, while also addressing associated biosafety and ethical concerns, calling for robust regulatory frameworks.

## Graphical abstract

The CRISPR/Cas system has emerged as a transformative tool in the field of molecular diagnosis, offering unprecedented sensitivity, specificity and rapid detection capabilities. This review further examines the molecular mechanisms of CRISPR tools, compares their clinical efficacy with traditional methods and highlights cutting‐edge innovations such as amplification‐free detection and AI integration.

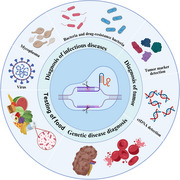

## 1 INTRODUCTION

Originally identified as a bacterial immune mechanism, the CRISPR/Cas system prevents viral infection by recognising and cleaving foreign DNA and has emerged as a revolutionary tool in molecular diagnostics. From its serendipitous identification in *Escherichia coli* genomes in 1987^1^ to the groundbreaking engineering of Cas9 as a programmable gene‐editing enzyme in 2012,^2^ this technology has redefined the boundaries of precision biomedicine. By 2016, the discovery of Cas12a's trans‐cleavage activity marked a pivotal shift towards its diagnostic applications, coinciding with global health crises such as the Ebola outbreak,^3^ which exposed the limitations of conventional PCR‐based methods in resource‐limited settings. This historical synergy underscores how technological innovation often emerges in response to pressing societal needs.

This review focuses on pathogens – including viruses, bacteria and fungi – encompassing both CRISPR‐based detection technologies and targeted anti‐pathogen strategies.

Traditional nucleic acid diagnostic techniques include culturing techniques, polymerase chain reaction (PCR),^4^, ^5^, ^6^ immunological assays and the microbial profiles, and microbial profiling.^7^ Although these conventional methods have facilitated the monitoring and identification of bacteria in real‐world samples, they often rely heavily on time‐consuming culturing and biochemical analysis.^8^ Additionally, these techniques require specialised equipment, facilities and skilled personnel, making them less accessible and cost‐effective, especially in resource‐limited settings.^9^ These limitations do not align with the World Health Organization's criteria for ideal diagnostic tools, which should be affordable, sensitive, specific, user‐friendly, rapid, equipment‐free and capable of providing real‐time results.

Emerging methods such as biosensors offer potential solutions but still face challenges in achieving the desired level of selectivity at a lower cost.^10^ Technologies based on molecular probes and fluorescence imaging, for instance, have been widely used in bacterial detection due to their non‐invasive nature, high specificity and sensitivity.^11^ However, issues related to ease of use, maintenance and real‐time in situ detection remain to be addressed.^12^ While the commercialisation of these sensors is currently limited, they hold significant promise for detecting pathogens in various environments.^9^

However, the translation of CRISPR diagnostics from controlled laboratories to real‐world environments reveals critical challenges. For instance, field studies in sub‐Saharan Africa highlighted a 63% performance drop in Cas14‐based assays under high humidity, emphasising the fragility of enzymatic activity in non‐ideal conditions.^13^

This review comprehensively examines CRISPR‐driven diagnostics – including viruses, bacteria and fungi – by exploring both CRISPR‐based detection technologies and targeted anti‐pathogen strategies. It further dissects the molecular mechanisms of CRISPR tools, compares their clinical efficacy with traditional methods and highlights cutting‐edge innovations such as amplification‐free detection and AI integration. Ultimately, we argue that CRISPR diagnostics must advance beyond technical refinement towards ecological adaptability, ensuring that precision medicine serves as a bridge – rather than a barrier – to global health equity.

## 2 MOLECULAR MECHANISM OF CRISPR/CAS SYSTEM AND ITS CLASSIFICATION

The CRISPR/Cas system is a powerful tool for gene editing, mainly utilising the process of homology‐directed repair (HDR),^14^ which is known for its high specificity and efficiency. Cas9 has been identified as a versatile and cost‐effective instrument in a variety of scenarios.^15^, ^16^ This advancement paves the way for the repair of clinically relevant mutations and improved nucleic acid detection. It has been demonstrated that several naturally occurring CRISPR nucleases, including Cas9, Cas12a (Cpf1) and Cas13a, have been repurposed for the detection of nucleic acids.^17^ It has been established that these nucleases possess high levels of both sensitivity and specificity.^18^, ^19^ They thus represent significant diagnostic tools for the identification of pathogens, including bacteria, viruses and parasites.^20^ Notwithstanding the aforementioned advantages, the technology is subject to certain limitations, including the paucity of comprehensive reference databases, the potential for off‐target effects and the challenge of distinguishing between living and dead organisms.^21^

The CRISPR/Cas system's versatility has enabled the development of innovative diagnostic platforms, including the Specific High Sensitivity Enzyme Reporter Unlocking (SHERLOCK)^22^ and the DNA Endonuclease Targeted CRISPR Trans Reporter (DETECTR).^3^, ^23^ These platforms utilise a variety of CRISPR enzymes to enable rapid and accurate nucleic acid detection. For instance, the Cas12a‐based DETECTR system has been demonstrated to detect trace amounts of DNA in samples, yielding results that are comparable to those of PCR‐based methods. The mechanism of Cas12a involves the recognition of its target DNA through the use of guide RNA (gRNA) and T‐rich in situ adjacent motifs (PAM),^24^ thus enabling the detection of a wide range of targets, including bacteria and viruses.

Despite the extensive utilisation of PCR for nucleic acid detection purposes,^25^ the technique is not without its inherent limitations. These limitations include, but are not limited to, reduced sensitivity, the requirement of sophisticated equipment and the necessity for trained technicians.^26^ Conversely, the CRISPR/Cas system offers a rapid, sensitive and specific method that overcomes these drawbacks.^18^ The combination of the CRISPR/Cas system with nucleic acid amplification techniques has been demonstrated to enhance detection sensitivity and specificity. This approach has been demonstrated to increase the abundance of target nucleic acids and to improve the specificity of the post‐amplification assay.^25^ Further advances have been made in the field, with the integration of CRISPR/Cas reagents into lyophilised formats, microfluidic microarrays and lateral flow assays. This development has enabled point‐of‐care diagnostics with minimal instrumentation.^24^ These developments have significantly expanded the application of CRISPR/Cas systems in diagnostics, establishing the foundation for future innovations in the detection and monitoring of infectious diseases. It has been demonstrated by a number of platforms, including SHERLOCK, DETECTR and One hour Low‐Cost Multipurpose Reporter (HOLMESv2),^27^ that CRISPR‐based assays have the potential to serve as an effective alternative to traditional methods. In the following discussion, the underlying mechanisms of CRISPR system detection will be examined, along with the methods and applications of each typology in detection, with a particular focus on bacterial detection.

### 2.1 Mechanisms of CRISPR/Cas system

In addition to its use in gene editing, the ability of the CRISPR/Cas system to recognise and detect specific DNA sequences extends its potential applications to bacterial detection. Through the exploitation of the activity of Cas proteins to precisely cleave DNA under the guidance of sequence‐specific CRISPR RNA (gRNA), researchers have developed a bacterial assay that is characterised by high specificity and sensitivity.^28^ This innovation creates new possibilities for diagnostic applications in healthcare and other fields.^21^ As demonstrated above, the mechanisms of target recognition and enzyme activity triggering have resulted in the development of various Cas protein‐based assays, each with a distinct function. The subsequent analysis will address these assays in relation to the types of targets and technical characteristics they possess.

#### 2.1.1 Target recognition: Complementary pairing of crRNA and target nucleic acids

The function of CRISPR RNA (crRNA) is to act as a guide molecule, with the capability of recognising specific sequences of target nucleic acids (DNA or RNA) through base complementary pairing. In its natural state, crRNA is transcribed and processed from the CRISPR motif and carries a spacer region sequence that is complementary to the exogenous invasive nucleic acid.^29^ When the CRISPR/Cas system is applied to diagnostics, artificially designed crRNAs can precisely target conserved regions of pathogen nucleic acids, such as bacterial *16S rRNA* genes or drug‐resistant genes, to achieve specific recognition.^30^ This targeting mechanism is highly programmable and can be adapted to different pathogens by modifying crRNA sequences.^31^

#### 2.1.2 Enzymatic activity trigger: Conformational change and trans‐cleavage of Cas proteins

Upon target recognition, Cas proteins undergo conformational changes that activate their nuclease activities, including cis‐cleavage (target‐bound nucleic acids) and trans‐cleavage (non‐specific cleavage of surrounding nucleic acids). Cas12, for instance, has been observed to induce collateral cleavage activity upon the recognition of target DNA, in addition to non‐specific trans‐cleavage of single‐stranded DNA (ssDNA).^3^ Similarly, Cas13 has been shown to activate trans‐cleavage of single‐stranded RNA (ssRNA) upon binding of target RNA.^22^ This trans‐cutting property is widely employed for signal amplification; for instance, it can be utilised in the design of fluorescent reporter probes that release fluorescent signals from the cutting probe when activated by Cas proteins. This enables the visualisation of nucleic acid detection.^32^

### 2.2 Comparative analysis of CRISPR tools

#### 2.2.1 Cas9

**FIGURE 1:**
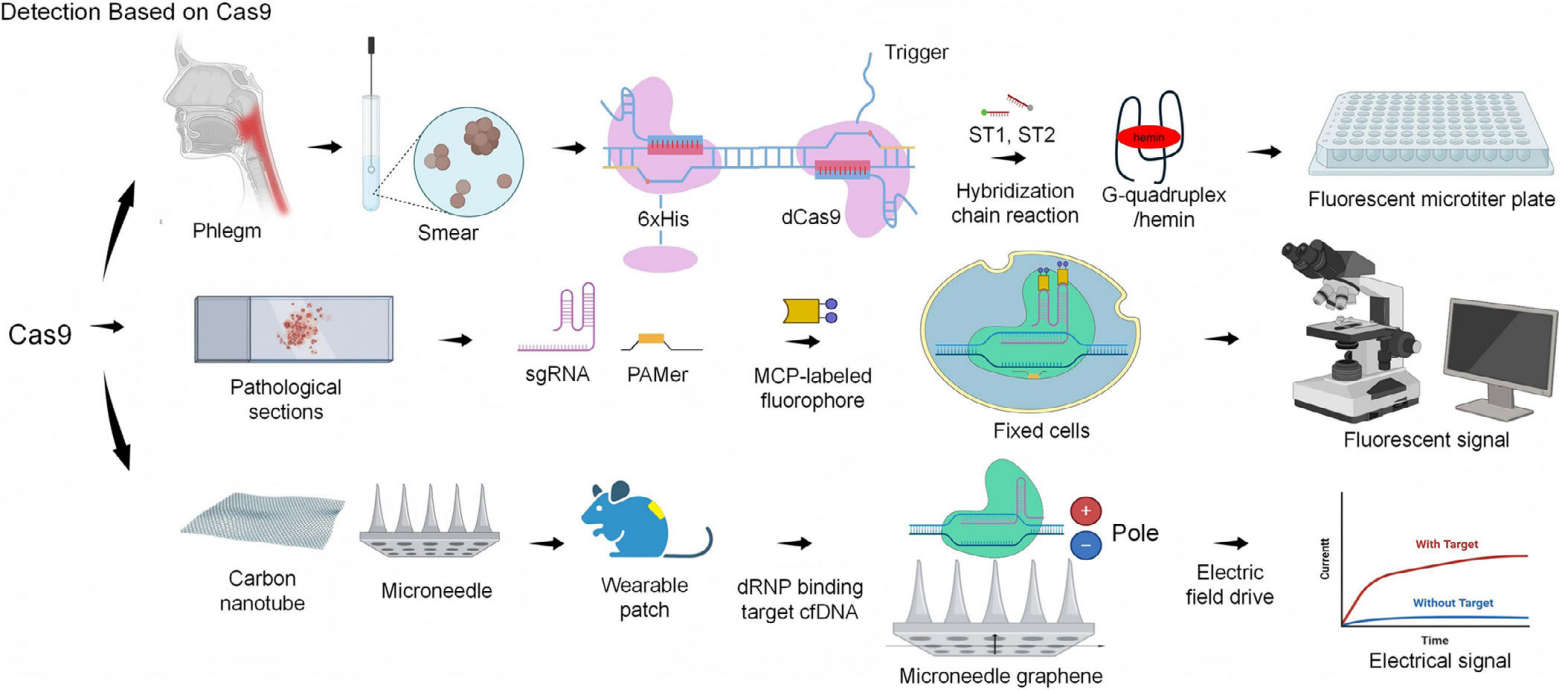
Detection technologies based on the CRISPR‐Cas9 system. The application of CRISPR/Cas9 in detection encompasses dCas9 detection using enzyme‐free amplification and HCR chromogenicity, as well as cellular in situ hybridisation using Cas9 specificity. Furthermore, graphene‐based wearable real‐time monitoring of target nucleic acid electrical signals is also possible.

**FIGURE 2:**
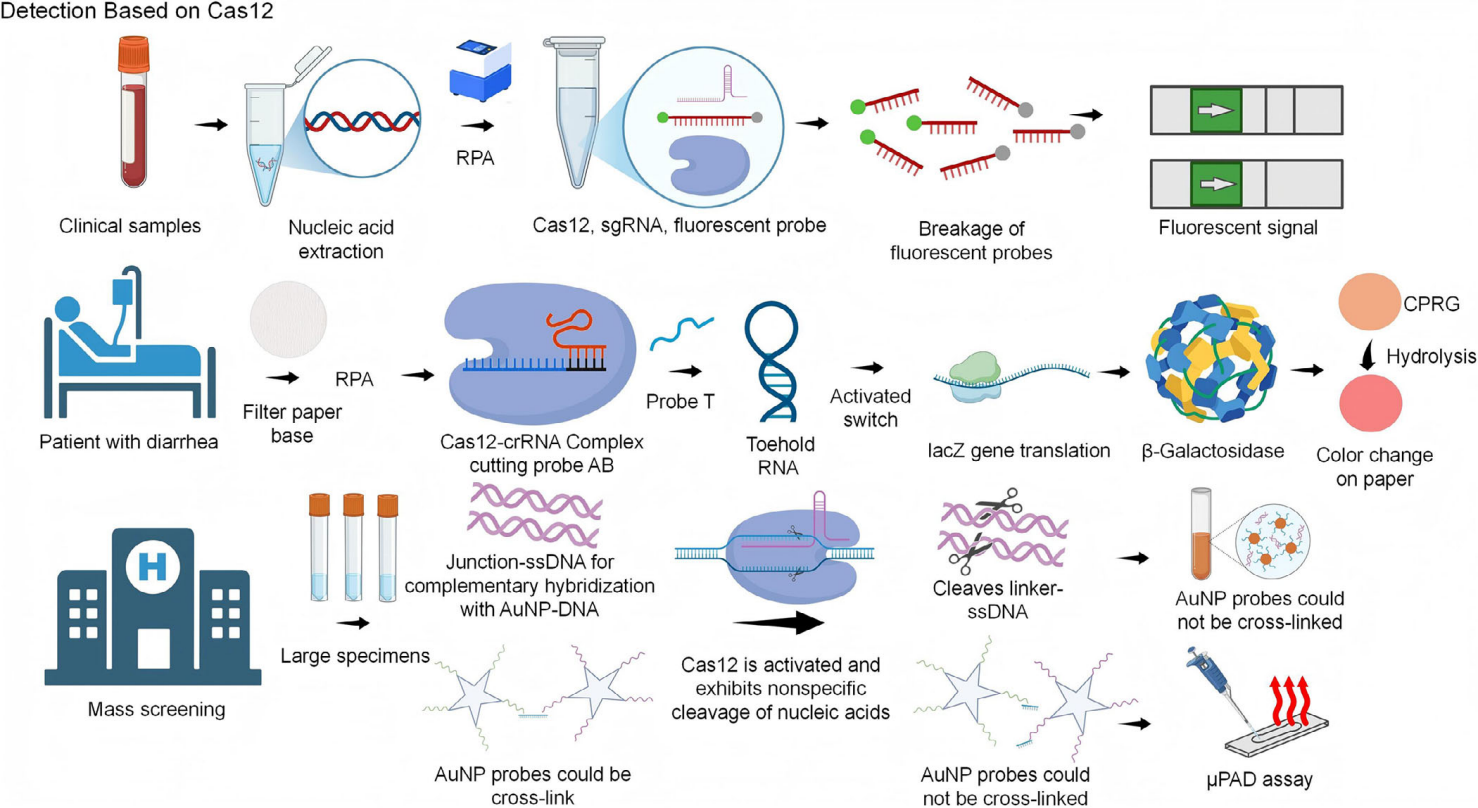
Detection technologies based on the CRISPR‐Cas12 system. The utilisation of CRISPR/Cas12 detection technology offers considerable potential for application in clinical samples and large‐scale detection, through nucleic acid amplification or direct detection on paper‐based substrates, or through the use of gold nanoparticles for rapid, efficient, and cost‐effective detection.

CRISPR/Cas9 systems have become versatile tools for nucleic acid detection, especially in the second class of CRISPR/Cas systems.^33^ The main advantage of CRISPR/Cas9 is its ability to accurately target any genomic site using programmable single‐guide RNAs (sgRNAs) for a range of applications such as genome editing, gene repair and targeted marker insertion, among others (Figure 2) (Figure 1).^34^ It is important to note that researchers are not required to possess expertise in the domain of protein engineering in order to achieve locus‐specific gene editing. This can be accomplished by utilising solely sgRNA and Cas9 enzymes, a method that renders the system both straightforward to operate and readily adaptable.^35^ However, despite the versatility of the system, there are limitations to its application in nucleic acid detection, such as the high cost of indirect signal generation and detection, as well as operational complexity, off‐target activity and dependence on other factors.^36^

The inherent inability of CRISPR/Cas systems to directly amplify nucleic acids or produce detectable signals necessitates their utilisation in conjunction with alternative amplification techniques or complementary sensing technologies.^37^ Nucleic acid amplification is a fundamental strategy for molecular diagnostics, and the combination of this process with PCR‐based methods offers a promising avenue for CRISPR/Cas‐based nucleic acid detection. For instance, the CRISPR/Cas9 typing PCR 4.0 (ctPCR4.0) method^38^ is a CRISPR/Cas9‐dependent PCR technique that employs the precise targeting capability of CRISPR/Cas9 to enhance the specificity of conventional PCR, thereby circumventing the limitations associated with primer specificity. The limitations associated with primer specificity are overcome. Furthermore, a methodology exists that enhances the detection capability by combining rolled loop amplification technology for *Staphylococcus aureus* detection, which has a detection limit of 7 CFU/mL.^39^

In addition to conventional nucleic acid amplification techniques, the integration of CRISPR/Cas9 with electrosensors has facilitated the development of innovative detection platforms. These platforms, including microneedle‐graphene‐based wearable systems, facilitate real‐time, non‐invasive monitoring of mutant genes^40^, ^41^ without the requirement for target or signal amplification. Furthermore, Cas9‐based diagnostics, including the FnCas9 Editorial Association Unified Detection and Analysis (FELUDA),^42^ which facilitates direct enzymatic detection of pathogen‐associated DNA/RNA sequences and single nucleotide variants, illustrate the relevance of CRISPR/Cas9 in point‐of‐care contexts. Notwithstanding the extensive potential of CRISPR/Cas9 technology for gene editing and diagnostics, certain limitations, including the formation of off‐target double‐strand breaks (DSBs), the toxicity of Cas9 and the low expression levels of Cas9 or sgRNAs, serve to limit its therapeutic applications.^43^ For example, CRISPR‐Cas9‐induced DSBs can lead to larger genomic rearrangements, including large chromosomal deletions, inversions or translocations and even more severe catastrophic events such as chromosomal loss, aneuploidy, chromosomal deletions and p53 activation, thereby increasing the number of cancerous cells. Other outcomes of CRISPR‐Cas9 editing include the integration of exogenous sequences, including lentiviruses, adeno‐associated viruses, plasmids and small DNA fragments, as well as LINE‐1 retrotransposons.^44^

The design of sgRNAs remains a pivotal factor in determining the efficiency of CRISPR/Cas9 RNP (ribonucleoprotein) systems, which poses a significant challenge in the development of effective detection systems. Nevertheless, advances in sgRNA design will provide a solid foundation for the future development of CRISPR/Cas‐based nucleic acid detection technologies.

Despite the evident potential of Cas9 technology in the domain of bacterial detection, it is imperative to acknowledge the inherent limitations of the technology, which are pivotal to its effective application and further development. Cas9 has been observed to exhibit off‐target effects, which have the potential to impact diagnostic accuracy, manifesting as false positives resulting from DSBs and design flaws in sgRNAs that can compromise specificity. Off‐target effects represent a significant concern in the field of translational medicine concerning Cas9. Research in this area has identified two primary factors contributing to these effects: firstly, the ‘lenient’ requirements of Cas9 for PAM sequences, which results in the formation of suboptimal binding sites, and secondly, the mismatch tolerance exhibited by Cas9 itself. In order to assess off‐target effects, a range of methods have been developed, including nuclease binding assays.^45^

Conventional detection methodologies reliant upon single‐gene recognition are susceptible to the occurrence of false positives, a phenomenon attributable to off‐target binding.^46^ By contrast, dCLISA employs a dual‐specificity recognition approach, utilising two dCas9/crRNA complexes that target the ‘r1’ and ‘r2’ regions of the *mecA* gene, respectively, thereby significantly mitigating the probability of off‐target binding.^46^ With regard to the optimisation of sgRNA design to reduce off‐target effects, related studies have indicated that existing sgRNA library rules are characterised by inflexible defects. The development of a Cutting Frequency Determination (CFD) score for off‐target site prediction, in conjunction with the utilisation of novel sgRNA design rules, has resulted in the optimisation of a sgRNA library.^47^

At present, the application of Cas9 technology in clinical settings is primarily focused on therapeutic applications, with only a limited number of examples of technologies related to detection, and even fewer examples of bacterial detection. Among cases involving the utilisation of CRISPR detection technology in clinical translation, the most salient example in recent years is the SHERLOCK detection technology case, which will be discussed in the following section.

Certain Cas9 technologies are already being utilised in the domain of gene therapy within clinical settings. A notable example is the in vivo gene editing technology NTLA‐2001.^48^ The technology has received FDA approval for an Investigational New Drug (IND) application and is currently undergoing related clinical trials. In a similar vein, for CRISPR/Cas‐based bacterial detection technologies to obtain relevant approvals, it is essential that the diagnostic technologies are managed within the three‐tier risk classification system and that the experience of other CRISPR technologies is drawn upon. For instance, adherence to the FDA's classification and management framework for in vitro diagnostic devices is imperative. It is imperative that technical requirements and compliance encompass explicit performance standards. Such standards should encompass analytical performance, clinical performance, diagnostic agreement rate and stability. Thirdly, it is imperative that the quality management system complies with FDA 21 CFR Part 801. For further information, please refer to the relevant documents published by the FDA.

#### 2.2.2 Cas12 (e.g. Cas12a, Cas12b)

In addition to Cas9‐based nucleic acid assays, the CRISPR/Cas12 system is gaining attention as an important tool for nucleic acid diagnostics, with a target type of DNA (activation of the trans‐cleavage activity of incidentally cleaved ssDNA after specific cleavage of the target DNA).^49^ The high sensitivity and specificity of the CRISPR/Cas12 system are attributable to its trans‐cleavage activity, short reaction time and wide range of applications in the field of biosensing. These characteristics position the CRISPR/Cas12 system as an ideal candidate for the development of simple and rapid assays (Figure 2).

The combination of Cas12 with isothermal amplification technologies (e.g. RPA, ERA, LAMP) has been demonstrated to enhance the sensitivity of the assay, enabling the detection of trace nucleic acids without the necessity for complex pre‐amplification. For instance, the RPA/CRISPR/Cas12a platform for *Pseudomonas aeruginosa* has been shown to have a detection limit as low as 100 copies/µL by fluorescence assay and 101 copies/µL by lateralised chromatography.^50^ In addition, the detection limit for *Mycobacterium tuberculosis* DNA can be as high as 100 copies/µL. The detection limit of *M. tuberculosis* DNA is reported to be as low as 4.42 pM, and the amplification‐free detection process can be completed within 50 min.^49^ In addition, the detection limit of SARS‐CoV‐2 nuclear coat protein is as low as 1 fg/mL by Cas12a‐mediated immunoassay, and the sensitivity is four orders of magnitude higher than that of traditional ELISA.^51^

Cas12 assays detect crude samples in ≤60 min, enabling rapid diagnostics in clinical, food and environmental settings. For instance, the presence of *Chlamydia pneumoniae* can be detected by the ERA‐CRISPR/Cas12a dual system. The results of this detection process are obtainable within 30 min for fluorescence and 15 min for lateral chromatography. Furthermore, the system exhibits no cross‐reactivity with seven other pathogens, thus ensuring 100% accuracy in the detection process when utilised on clinical samples.^31^The detection of *Salmonella* on a paper substrate is achieved through a combination of RPA (reverse transcription‐PCR) Cas12a. The differentiation of *Salmonella typhimurium* and *Salmonella enteritidis* is possible without the need for redesign of probes, and the detection limit is as low as 100 genome copies. This method has been successfully applied to milk and lettuce contaminated samples.^52^ The utilisation of microfluidic microarrays in the detection of respiratory viruses required less than 40 min, while the detection of sugarcane streak mosaic virus in crude plant leaf extracts was accomplished in 50 min. This outcome demonstrates the applicability of the method in field and primary care contexts.^53^, ^54^ These technologies demonstrate the high efficiency of Cas12a in detection, offering the possibility of more efficient bacterial detection.

Cas12 facilitates the concurrent identification of multiple pathogens within a single tube or microarray platform, such as a dual system integrating Cas12a (DNA detection) and Cas13a (RNA detection) for the simultaneous detection of maize Stewartia (bacteria) and maize dwarf mosaic virus, with detection limits of 1 × 10^−6^ ng/µL and 3.69 × 10^−7^ ng/µL, respectively.^55^ In the future, it is anticipated that multiple bacterial detection platforms will be constructed based on similar principles. The RPA‐CRISPR/Cas12a platform has been shown to specifically identify *Klebsiella pneumoniae* from a range of 11 clinical pathogens by means of fluorescence (1 fg/µL) and lateral chromatography (10 fg/µL) dual readout with 100% sensitivity and specificity for clinical samples.^56^ Furthermore, gold nanoparticle‐based CLAP technology and DISC2 (5) colorimetric assay have expanded the application of Cas12 for multiplexed detection and visualisation.^32^, ^57^

In order to enhance the performance of Cas12, researchers have focused on improving system components. This has included the use of dual synthetic mismatch CRISPR/Cas12a (dsmCRISPR) and AIOD CRISPR technologies, which utilise dual PAM site crRNAs with a view to reducing cost and increasing specificity.^58^, ^59^ Integrated electrochemical biosensor (E‐CRISPR) and isotropic electrophoresis technologies have been shown to be more sensitive than conventional fluorescent assays and do not require labelling. These technologies provide instrument‐free solutions for point‐of‐care testing (POCT).^60^, ^61^ While current technologies rely on amplification and fluorescent labelling, future studies are exploring direct detection methods that do not require amplification. For instance, electrochemical biosensors coupled with Cas12a have been used to further streamline the detection process through visual observation or smartphone interpretation of results.^62^

Concerns have been raised regarding the efficacy of Cas12‐based detection technologies. These concerns primarily encompass three areas: the initial signal conversion efficiency, the activation process and the interference of background signals. Additionally, issues have been identified with the stability of signal molecules and the interference of the matrix in complex samples.^63^, ^64^ Among these, non‐specific trans‐cleavage is a pivotal factor affecting the accuracy of Cas12 detection.^60^ It has been hypothesised that the generation of false positives may be associated with the background of Cas12 itself, variations in protein activity between batches, which may result in the cleavage of signal nucleic acids even in the absence of a target, and the binding of recognition elements to other substances in the sample. This hypothesis is supported by related studies.

Given the strong technical compatibility and inherent advantages of Cas12 detection technology, it is theoretically feasible to develop a Cas12 detection system similar to PCR that targets specific bacterial nucleic acids, as well as specific bacterial proteins or glycoproteins. This would enable these technologies to better advance towards clinical translation. For example, developing universal paper‐based portable test strips or low‐cost reagent kits to facilitate testing in remote areas can adapt to different testing scenarios. During the process of advancing the clinical translation of these technologies, regulatory oversight must clearly define risk stratification while establishing dynamic communication mechanisms to address issues such as how to quantify cross‐reactivity in multiplex testing. Clinical validation of the technology requires scientific design, including multi‐centre data collection and validation in complex scenarios. Intellectual property issues and clear commercialisation pathways must also be addressed. Relevant case studies can draw on Mammoth Biosciences' LAMP‐Cas12a cascade reaction technology for tuberculosis detection. The company is also advancing its CRISPR/Cas14‐based detection technology, whose characteristics will be elaborated upon in subsequent discussions.

In conclusion, the CRISPR/Cas12 system, with its ultra‐high sensitivity, rapid detection and multi‐adaptation capability, has demonstrated significant advantages in the detection of bacteria, viruses and other pathogens. The portable platform combined with isothermal amplification technology provides an efficient solution for primary healthcare, food safety and environmental monitoring.

#### 2.2.3 Cas13 (e.g. Cas13a)

Cas13 is an RNA‐inducible RNase consisting of four variants: Cas13a, Cas13b, Cas13c and Cas13d. Its structural characteristics are defined by the presence of two higher eukaryotic and prokaryotic nucleotide (HEPN)‐binding domains, which facilitate the cleavage of ssRNA molecules. Cas13's unique collateral cleavage activity allows it to target and cleave non‐specific RNA around the intended RNA target.^65^ This distinctive property renders Cas13 a potent instrument for RNA‐based nucleic acid detection.

**FIGURE 3:**
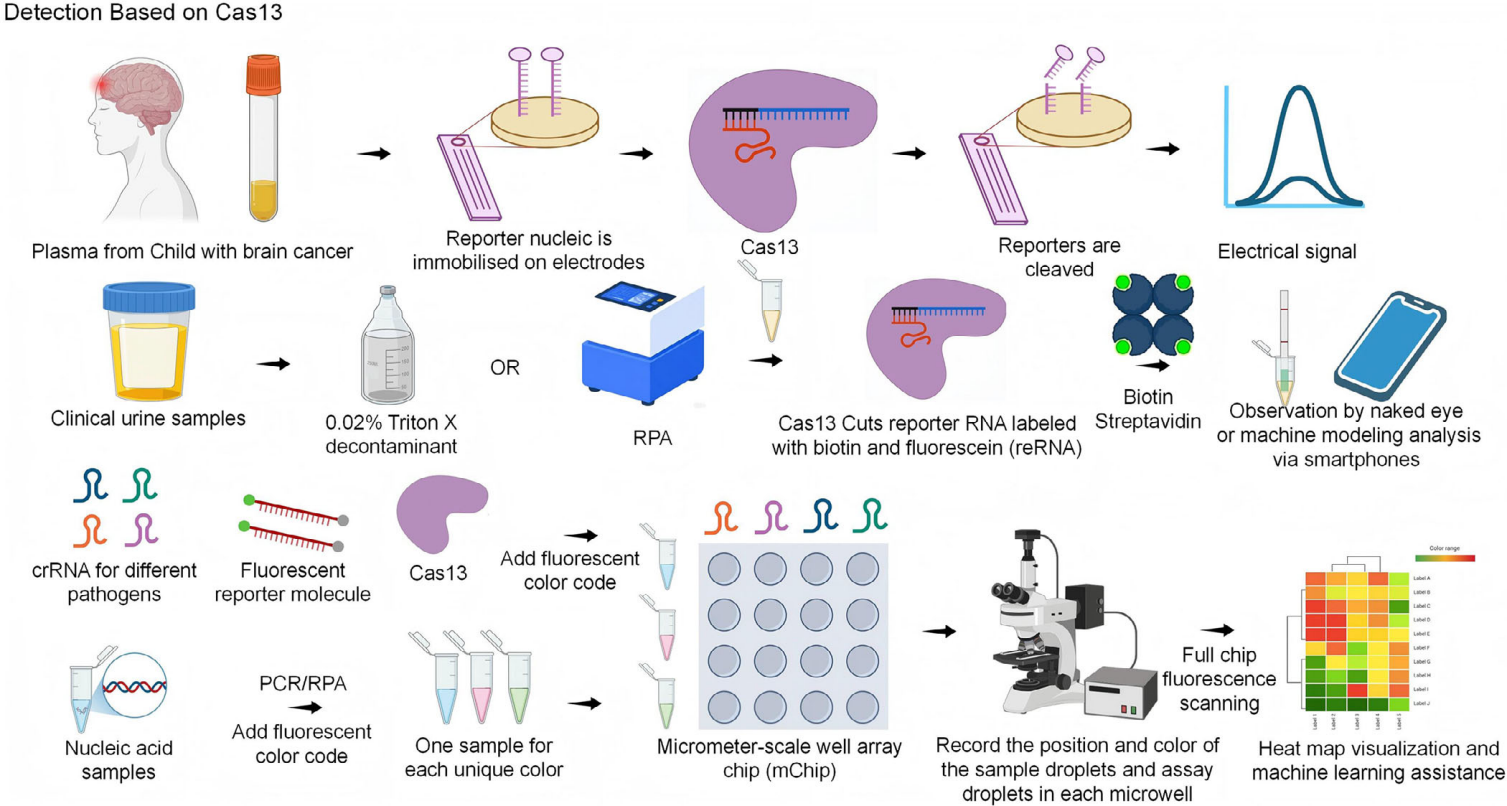
Detection technologies based on the CRISPR‐Cas13 system. The utilisation of CRISPR/Cas13 in detection encompasses a range of immunology‐based methodologies, including antibody fixation and electrical signal binding, isothermal amplification, and lateral flow paper strip readout. Additionally, it involves micrometre‐scale well array chip (mChip)‐based oversized samples and multiplexed pathogen detection methods.

The development of Cas13‐based technologies has become a significant focus in the domain of nucleic acid testing, particularly in the context of RNA‐targeted diagnostics. While the aforementioned strategies bear similarities to the CRISPR/Cas9 and Cas12 systems, often requiring integration with nucleic acid amplification techniques, Cas13 specifically targets RNA and therefore requires additional steps. These include reverse transcription of the RNA virus to DNA, followed by amplification and transcription back to RNA for detection.^66^ Cas13 enables single‐tube detection of RNA viruses (e.g. avian influenza viruses, dengue viruses) by integrating isothermal amplification techniques. For instance, in the detection of H5/H7/H9 subtypes of avian influenza viruses, the Cas13a platform combined with recombinase polymerase amplification (RPA) has been shown to have a detection limit of 1 copy/µL (fluorescence assay), with the process being completed within 60 min and visualised by lateral flow test strips. As demonstrated in Figure 3, the detection limit for the identification of dengue viruses was determined to be 91.7 copies of the RPA in combination with Cas13a, and 91.8 copies of the RNA of dengue viruses. The dengue virus was detected by RT‐RPA in combination with Cas13a, with a detection limit of 91.7 copies/reaction in 40 min.^67^ The novel virus and its mutants were identified using the following methods: SHERLOCK,^22^, ^68^ SHINE^69^ and ADESSO.^70^ As demonstrated in references and other classical platforms, the highly specific recognition ability of Cas13 enables the highly sensitive detection of viral genomes, novel variants and single nucleotide mutations. Furthermore, some of the methods are capable of detecting trace RNA without the need for pre‐amplification.

The integration of Cas13 technology with visualisation techniques and portable devices has led to a substantial enhancement in the field's capacity for virus detection. For instance, the RPA‐CRISPR/EsCas13d platform, developed for pseudorabies virus, integrates RPA with the trans‐cutting activity of Cas13d on a dual‐readout portable platform, achieving a detection limit of as low as 1 copy/µL. The platform supports side‐by‐side chromatography strips or fluorescent visualisation, enabling the completion of detection in less than 1 h with quantitative real‐time PCR (qPCR) results. It is considered suitable for pig farming and public health monitoring in remote areas without complicated equipment.^71^ Furthermore, a smartphone‐based machine learning application for *Neisseria gonorrhoeae* detection provides a rapid diagnostic solution for primary care providers without the need for specialised instruments by using machine learning algorithms to assist in the interpretation of lateral flow test strip results, with 100% accuracy in clinical urine samples, and with an optimised response time of 60 min.^72^

The integration of expression monitoring of carbapenem‐resistant genes with the RPA‐Cas13a system facilitates a molecular level of support for real‐time assessment of bacterial drug resistance with attomolar detection limits and bedside diagnosis in less than 50 min.^73^

The combination of Cas13 with electrochemical biosensors and digital bioassay technologies serves to extend the functionality of Cas13, thereby enabling efficient and rapid detection with minimal amplification. For instance, the CRISPR‐based amplification‐free digital RNA assay (SATORI)^74^ platform integrates Cas13‐based RNA detection with microcompartmental array technology, while the DEX droplet system^75^ enables digital detection of SARS‐CoV‐2 RNA. In a similar manner, the Whole Genome Nucleic Acid Chromatography Surveillance (WATSON) assay utilises Cas13 technology to detect bacterial cell‐free deoxyribonucleic acid, including that derived from *M. tuberculosis*, directly from patient plasma.^76^

Advancements in Cas13 technology have also facilitated simultaneous detection and high‐throughput analysis of multiple targets. The Combined Array Reaction for Multiplexed Evaluation of Nucleic Acids (CARMEN) platform, when employed in conjunction with Cas13 (CARMEN‐Cas13), facilitates the robust detection of thousands of target pairs on a single array, thereby significantly reducing the cost of detection and supporting high‐throughput screening. This capacity is of particular importance for comprehensive pathogen surveillance and public health monitoring.^77^

In addition to the detection of pathogens, Cas13 has been employed in the domain of live cell imaging and gene expression analysis. The live cell fluorescence in situ hybridisation (LiveFISH) technique employs Cas13 to monitor RNA and chromosome dynamics in living cells. This method employs fluorescent oligonucleotides to visualise RNA transcripts and genomic DNA in real time, thereby providing insight into gene expression and chromosomal disorders. Furthermore, Cas13 can be utilised for single‐cell sequencing to target RNA perturbation. CaRPool‐seq (Cas13 RNA Perturb‐seq) is one such method that enhances the accuracy and efficiency of combinatorial single‐cell gene perturbation studies.^78^

The potential of Cas13 technology in the field of bacterial diagnostics has yet to be fully realised. Future research could focus on optimising RNA extraction efficiency, reducing background signal interference and developing more convenient amplification‐free assays. The development of isothermal amplification technology, microfluidic chips and smartphone reading technology has led to the prediction of a significant increase in the use of Cas13 in a variety of applications, including the detection of RNA viruses, the monitoring of drug‐resistant genes and the diagnosis of bacterial infections. This is particularly relevant in the context of preventing and controlling infectious diseases in resource‐poor areas, where Cas13 is considered to be highly advantageous due to its convenience and efficiency.

It is inevitable that Cas13, as part of the CRISPR/Cas detection technology, will encounter issues of non‐specific detection. A number of relevant studies have revealed that the collateral cleavage of activated Cas13^22^ interferes with the detection of low‐abundance RNA. This interference is primarily characterised by two distinct manifestations: firstly, a disruption in the accuracy of detection; and secondly, an association with detection sensitivity. Research conducted on off‐target effects has demonstrated that the target RNA binding affinity activity of Cas13a is influenced by the number and location of mismatches between crRNA and target RNA.^79^ Cas13a has been demonstrated to exhibit a degree of tolerance to single‐nucleotide mismatches between crRNA and the target sequence,^80^ suggesting a capacity for non‐target RNA recognition during the detection process.

Cas13a's collateral cleavage activity is only activated after the formation of the crRNA‐target RNA double strand,^81^ thereby ensuring against inaccurate cleavage. In order to circumvent the occurrence of false‐positive detection results that have been attributed to non‐specific cleavage activity, relevant experiments have achieved the absence of cross‐reactions caused by non‐targeted cleavage by employing a precise design of crRNA.^82^ In order to enhance the accuracy and reliability of the results obtained from the reaction system, it is essential to minimise background interference. This can be achieved by optimising the reaction buffers and enzyme concentrations, thereby improving the signal‐to‐noise ratio.^22^

The most clinically relevant example of CRISPR/Cas13‐based detection technology is the SHERLOCK technology. As of July 2025, the company responsible for SHERLOCK has made significant progress in the commercialisation of CRISPR‐related detection technologies, including technology licensing and collaboration, patent strategy and technology extension. The company has advanced comprehensive clinical trials and submitted an FDA application to achieve clinical translation by leveraging its technological advantages.

In addition to SHERLOCK's FDA certification application, other relevant cases include Sense Biodetection's VerosCOVID‐19 test, which has undergone multi‐centre clinical trials,^83^ variant strain inclusivity validation, CE certification modular design and clinical data support to achieve CE certification. The approval of related technologies in Europe necessitates a consideration of the EU's policy support for different disease detection methods. It has been established that Sherlock has entered into a global patent cross‐licensing agreement with ToloBio, which encompasses the utilisation of Cas12/13 technology. Presently, the National Medical Products Administration of China is undertaking measures to enhance the approval process for innovative medical devices. This development has the potential to streamline the entry of CRISPR‐related technology products such as SHERLOCK into the Chinese market.

#### 2.2.4 Other Cas variants and emerging tools

Cas14a1 overcomes the dependence of traditional CRISPR on PAM sequences by single‐primer isothermal amplification of integrated biosensors (SPCas) to achieve ultra‐sensitive detection of *Salmonella typhi*. The technology utilises RNA–DNA primers and chain substitution reactions to activate the trans‐cutting activity of Cas14a1, with a detection limit as low as 5 CFU/mL and 100% diagnostic accuracy in clinical samples.^13^ Furthermore, the Cas14VIDet platform obviates the necessity for PAM sequences, while concomitantly accomplishing *H. pylori* drug resistance gene detection within a timeframe of 10 min, with a sensitivity that approaches the level of a single bacterial colony (100 CFU/mL).^84^

**FIGURE 4:**
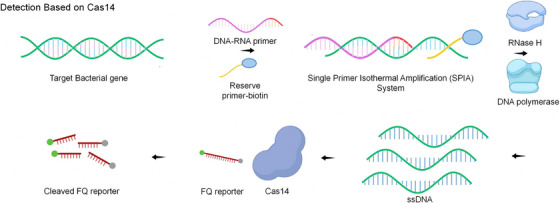
Detection technologies based on the CRISPR‐Cas14 system. The employment of CRISPR/Cas14 in the detection process, in conjunction with the single primer isothermal amplification (SPIA) system, facilitates the identification of target pathogen nucleic acids.

Furthermore, the Cas14a1‐mediated pathogen nucleic acid detection platform is employed. As demonstrated in Figure 4, the detection limit of *Streptococcus pyogenes* and *E. typhimurium* is as low as 10^3^–10⁴ CFU/mL. The method has been shown to accurately differentiate the target bacteria in milk samples, with strong resistance to matrix effects. It is also able to differentiate target bacteria in milk samples and is resistant to matrix interference, providing a rapid test solution for food safety and infectious disease diagnosis without the need for complex equipment.

The Cas14a universal bacterial diagnostic platform^85^ combines a label‐specific primer extension and a magnetic DNA system, thereby increasing the sensitivity of the test to 1 CFU/mL (bacteria) and 1 aM (DNA). It is capable of distinguishing six pathogens simultaneously and is compatible with complex clinical samples, such as blood and urine. The integrated process simplifies the operation. The device has been demonstrated to be suitable for rapid on‐site screening of multiple pathogens and has significant potential for application, especially in areas where resources are limited.

CasX, a constituent of the V‐type CRISPR system, possesses a compact protein structure and flexible PAM recognition properties, rendering it suitable for the development of miniaturised detection tools. Despite its current paucity of research, the potential of this method for the detection of Gram‐negative bacteria has been explored in preliminary studies, and it is conceivable that it may become a fundamental component of portable detection platforms in the future.^28^

In recent studies, CasX has demonstrated the potential for application in the domains of highly specific bacterial typing, the detection of drug‐resistant bacteria and portable testing. The process of DNA cutting requires a minimum of 17 PAM proximal nucleotide matches to initiate stable binding, thereby enabling the detection of pathogens with a high degree of specificity. This is achieved by designing sgRNAs that target drug‐resistant genes (e.g. the bla family) or germline markers (e.g. 16S rRNA).^86^

The advantage of CasX in terms of its small size renders it suitable for use in the construction of portable detection platforms, in conjunction with nanoparticles, microfluidic chips and other vectors. Optimisation of sgRNAs (e.g. sgRNAv2) to enhance their binding stability to nucleic acids,^87^ in combination with lateral flow chromatography or fluorescence technology, enables rapid detection of bacteria in clinical or food samples. For instance, the cleavage activity of the restriction endonuclease can be exploited to develop a PCR‐free assay capable of detecting Listeria monocytogenes in milk within 30 min.^88^ In the future, the utilisation of CasX's sensitivity to PAM distal mismatches has the potential to facilitate the design of multi‐sgRNA combination probes. This approach would enable the simultaneous detection of multiple targets while concomitantly reducing the false‐positive rate. When combined with single‐cell analysis and real‐time fluorescence imaging, monitoring its cleavage signal (S1→S3) has been shown to resolve the single‐cell drug resistance status and provide guidance for individualised treatment.^88^ Despite its comparatively diminished target search efficiency in comparison to Cas9 and Cas12a, the potential exists for enhancement of detection speed through the utilisation of protein engineering or combined isothermal amplification technology in future studies. The development of ‘molecular switch’ type sensors based on conformational dynamics is expected to promote their field applications in food safety and environmental monitoring.

Cas3, the core protein of the type I CRISPR system, has been demonstrated to possess the ability to target deconvolution and cleave double‐stranded DNA. The primary applications of this technology are in the domains of gene editing and the degradation of pathogen genomes. In the context of bacterial detection, it can be employed in conjunction with nucleic acid enrichment techniques to facilitate the efficient capture of low‐abundance pathogen DNA. However, the prevailing focus of current research endeavours pertains to the analysis of its underlying mechanism, with the exploration of its potential applications in clinical detection remaining in its nascent stages.^29^

In context of Cas3‐related research, Type I‐A has exhibited favourable performance in the domain of detection, for instance. The HASTE assay platform is an innovative technique that utilises the ability of Cas3 to non‐specifically cleave nearby ssDNA reporter molecules following binding to the target DNA. This platform enables highly sensitive detection of dsDNA/ssDNA down to 1 pM, ssRNA at 0.1–1 nM and ssRNA at 0. The concentration of 1–1 nM is to be used in conjunction with the quantification of fluorescence and the testing of lateral flow chromatography strips. The ssRNA is to be activated by heat in a manner that is compatible with the processing of high‐temperature samples. The multi‐form detection supports the quantification of fluorescence and the testing of lateral flow chromatography strips. The low background detection is to be used to avoid false positives. Its potential applications include pathogen detection and multiplex testing.^89^

The CRISPR/Cas system has evolved to encompass a variety of detection tools, each distinguished by its distinct mechanism of target recognition and enzyme activity triggered by different Cas proteins. Among these, Cas9 functions as an auxiliary element in the detection of drug‐resistant genes, while Cas12 and Cas13 have emerged as the predominant technologies for the detection of DNA and RNA pathogens, respectively. Furthermore, the emergence of variants such as Cas14 has transcended conventional limitations, paving the way for novel PAM‐free detection and ultra‐sensitive diagnostic methodologies. The synergistic development of these tools has led to significant advancements in bacterial detection technology, resulting in rapid, accurate and portable detection methods.

## 3 SYSTEMATIC COMPARISON BETWEEN CRISPR/CAS AND TRADITIONAL BACTERIAL DETECTION TECHNIQUES

Table 1 provides a brief overview of several rapid detection technologies based on the CRISPR/Cas system. These technologies are capable of detecting bacteria such as *S. aureus* and *Salmonella*, viruses such as SARS‐CoV‐2, *noroviruses*, fungi and parasites, as well as other pathogens. The combination of CRISPR/Cas (e.g. Cas9, Cas12a and Cas13a) and amplification methods (e.g. RPA, RAA, LAMP and analogous techniques) has been shown in recent studies to achieve the goals of high sensitivity, speed and portability. These techniques are considered to be applicable to clinical, food safety and field‐testing scenarios. It is evident that certain technologies have been used in the development of commercial kits or for immediate diagnosis in resource‐limited settings.

**TABLE 1:** Comparison of CRISPR‐Cas detection technologies in clinical and field applications.

Technology	Target	Limit of detection	Detection time	Clinical scenario	Advantages	Limitations	Cost and feasibility	Suitability for clinical application	References
CRISPR/Cas9‐RPA	*Staphylococcus aureus*	10^2^ CFU/mL	2 h	‐	High sensitivity	Requires complex instrumentation, difficult primer design	$5000–8000, specialised equipment required	Laboratory research scenarios	^191^
CRISPR/Cas12a‐*n*anophotonic holographic	*Salmonella*	38 CFU/mL	1 h	Food/clinical samples	High sensitivity, label‐free	Reliance on pre‐amplification step, risk of cross‐contamination	Thermostatic equipment about $800–1200, reagent cost $120–200	Rapid laboratory and field screening	^137^
CRISPR/Cas12a‐HCR	*S. typhimurium*	77 CFU/mL	<1 h	Foodborne pathogen detection	High accuracy	Steps are cumbersome and require multiple levels of response	$1500–2000, professional operation required	Quality control in the food industry	^192^
CRISPR/Cas12a‐RT‐RPA	‐	1 copy/µL	<30 min	On‐site testing	Simplified operation				^193^
	*Norovirus GII.2*	10 copies/µL	35 min	On‐site rapid detection	Low cost	Requires reverse transcription step, limited sensitivity	$15–25, portable equipment	Primary care facilities and outbreak sites	^194^
CRISPR/Cas12a‐LAMP	SARS‐CoV‐2	6.2 × 10^2^ copies/mL	1.5 h	Commercial kit development	High specificity	Complex primer design, prone to aerosol contamination	Thermostat equipment about $1000–1500, kit cost about $20/time	Routine hospital nucleic acid testing	^195^
CRISPR/Cas12a‐RAA	*NiV*	10 copies/µL (fluorescence), 100 copies/µL (lateral flow)	‐	Rapid diagnosis in resource‐limited areas	‐	Large difference in sensitivity between fluorescence and test strips	$10–15, two types of testing equipment required	Hierarchical diagnostics for remote areas	^196^
	*Salmonella*	‐	30 min	‐	‐				^197^
CRISPR/Cas12a‐RPA	HPV16/18	6 copies/µL	36 min	Remote areas	Portable, high sensitivity, no cross‐contamination	Strict anti‐pollution measures	$25–40, portable equipment	Cervical cancer screening mHealth	^198^
	*B. cereus*	gDNA: 10⁻^2^ ng/µL; viable cells: 10^2^ CFU/mL	‐	Field screening in food	No complex equipment required	Difficulty in distinguishing between live and dead bacteria	$7–10, low cost, easy to operate	Food Processing Site Inspection	^199^
	*Streptococcus*	10 copies/µL	33 min	Point‐of‐care testing	High specificity				^112^
	*Fungal keratitis*	‐	50 min	Point‐of‐care testing	Fast, high sensitivity/specificity				^200^
	*Stenotrophomonas maltophilia*	3 copies/µL	30 min	On‐site testing	Avoids cross‐contamination, high sensitivity				^201^
CRISPR/Cas13a‐RAA	Bovine leukaemia virus (BLV)	1 copy/µL	100 min	BLV screening in farms	High sensitivity/specificity	Reagents requiring cold chain transport	$18–28, need to stabilise supply chain	Livestock herd monitoring	^202^
	*ALV‐J*	10^2^ copies	85 min	Early monitoring in farms	High sensitivity/specificity				^203^
CRISPR/Cas13a‐RPA	*Brucella BCSP31 gene*	100 copies/µL	‐	On‐site detection in clinics/pastoral areas	High sensitivity, portable				^204^
	*Nosema bombycis*	1 copy/µL	40 min	Silkworm industry testing	100% specificity, high sensitivity				^205^

### 3.1 Comparative analysis of performance parameters

#### 3.1.1 Sensitivity and specificity characteristics

Among molecular diagnostic technologies, CRISPR technology has been demonstrated to exhibit significant advantages in terms of sensitivity,^90^ with a detection limit of aM level. This enables the detection of SARS‐CoV‐2 RNA at 4 copies/µL^91^ and *S. aureus* at 5 CFU/mL.^92^ It has been demonstrated that the method exhibits a high degree of specificity,^93^ with the capacity to differentiate between *Cryptococcus subtypes* with 100% accuracy.^94^ Furthermore, it has been shown to accurately detect drug‐resistant genes,^95^ a feature that renders it invaluable in trace samples,^96^ such as wastewater monitoring.

In contrast, the qPCR technique exhibits a pM‐level sensitivity (∼10^3^ copies/µL) and maintains a specificity in the 95%–98% range, rendering it particularly well suited for routine nucleic acid quantification in a laboratory setting.^97^

Despite its lower sensitivity (≥10^3^ CFU/mL),^98^ the traditional culture method remains the gold standard for pathogen isolation and drug sensitivity testing due to its 100% phenotypic identification accuracy^99^ (a process which takes 24–72 h to complete). The physicochemical parameters that are of the utmost importance for the culture of a given pathogen vary according to the microorganism in question. For instance, *S. aureus* is cultivated on nutrient agar (pH 7.2–7.4) at 37°C under aerobic conditions; *Salmonella*, on the other hand, requires selective media (pH 7.4) and incubation at 35–37°C for 24–48 h. *Anaerobic bacteria* ^100^ require an oxygen‐free environment (containing ≤1% O_2_) and reduced media.^101^

Conversely, ELISA techniques with detection limits at the ng/mL level, such as aflatoxin B1 with a sensitivity of 10 pM,^102^ combined with 90%–95% specificity, balance cost and efficiency, and are the tool of choice for rapid food safety screening.^103^ The physicochemical parameters for ELISA include incubation of the antigen‐antibody at 37°C (optimal for immune complex formation); the use of a washing buffer (PBS with 0.05% Tween‐20, pH 7.4) to reduce non‐specific binding; and the use of a substrate reaction at 25–37°C with a pH of 4.5–5.0 for colour development, terminated by 2 M H_2_SO_4_.^104^

A comparison of CRISPR technology with next‐generation sequencing (NGS) and microfluidics reveals the distinctive characteristics of the former. NGS has been demonstrated to be capable of detecting multiple pathogens in high‐throughput settings. However, its sensitivity is limited when it comes to low‐abundance pathogens, such as SARS‐CoV‐2, where a cycle threshold (Ct) value greater than 35 is required for detection. To enhance its diagnostic accuracy to 100%, the incorporation of CRISPR pre‐processing is necessary.^105^ In contrast, CRISPR demonstrates superior performance in the analysis of trace samples, achieving an aM‐level detection limit without the need for complex library preparation. It has been demonstrated that CRISPR performs better in trace samples with aM‐level detection limit, without complex library preparation and with 100% specificity,^94^ while NGS requires bioinformatics analysis to eliminate interference and has 99% specificity.^106^ The pure microfluidic platform has the capacity to detect multiple targets (e.g. eight respiratory pathogens) in parallel, with a sensitivity of 10 copies/µL, and can produce results within 45 min,^106^ but the cost of the chip is high ($10–20).The CRISPR‐microfluidic integrated system has the capacity to amplify the signal of Cas protein, with a sensitivity of 1 copy/µL, and the cost of a single test is only $2–5 for a portable device.^93^

Table 2 systematically compares the core performance parameters and application scenarios of 13 molecular diagnostic technologies. The assays encompass gene sequencing, FISH, ELISA, PCR and its derivatives such as qPCR and digital PCR, isothermal amplification technologies such as LAMP and RCA, biosensors, NGS and microfluidics. These assays are compared in terms of detection limit, time‐consuming, cost, clinical applicability and other dimensions. Conventional methods, such as ELISA and PCR, have a detection limit of 105 CFU/mL and a rapid detection capability of 20 min, respectively, and are suitable for routine laboratories. Conversely, gene sequencing, although it can obtain high‐resolution genomic information with a detection limit of 1–10 ng/µL, is costly and time‐consuming, and is limited to research use. Emerging technologies such as digital PCR and conductivity biosensors have been shown to excel in sensitivity and speed, respectively. Digital PCR has been demonstrated to have a detection limit of 3.8 copies per 10⁵ cells, while conductivity biosensors can complete the assay in less than 10 min. However, it should be noted that both of these technologies rely on sophisticated equipment and high costs. Isothermal amplification techniques, such as LAMP, which boasts a thermostatic reaction and low cost, have a reaction time of less than 35 min and show potential in resource‐limited areas. However, they carry a risk of contamination. In clinical applications, RT‐PCR techniques such as those utilised for the detection of SARS‐CoV‐2 (hereafter referred to as ‘COVID‐19’) and qPCR are widely adopted due to their quantitative analysis capabilities, while FISH relies on probe design for specific diagnostics. Notwithstanding the fact that NGS can analyse the whole genome without bias and achieve large‐scale screening and accurate quantification, the process is time‐consuming, often taking hours or even days, and costly. Furthermore, it relies on specialised laboratories, which makes it difficult to meet immediate needs. Microfluidics, as a platform technology, is often used in conjunction with CRISPR, which can integrate the steps of sample processing to improve efficiency and reduce contamination. However, it does not have its own detection specificity and relies on conjugation technologies.

**TABLE 2:** Performance comparison of other pathogen detection technologies.

Technology	Target	Limit of detection	Assay time (min)	Applications	Disadvantage	Cost and feasibility	Suitability for clinical application	References
Gene sequencing	*16S rRNA* gene or whole genome	1–10 ng/µL	In a few days	The acquisition of high‐resolution information	Costly, complex to analyse, time consuming	High cost, requires specialised equipment	Research only, not clinical	^206^
FISH (fluorescence in situ hybridisation)	Specific genes or genome, *16S rRNA* gene	10^3^–10⁴ CFU/mL	In a few hours	Visualisation, observe bacteria under a microscope	Depends on probe design, complexity, and operational requirements	Medium cost, fluorescence microscope required	Specific diagnostics	^207^
ELISA	*Escherichia coli*, *Campylobacter fetus*, *Salmonella*, *Clostridium monocytogenes*	10⁵ CFU/mL	In a few hours	Detection of *Escherichia coli*, *Campylobacter fetus*, *Salmonella*, *Clostridium monocytogenes*	Multiple hours and steps required to complete the test	$5–10, using a kit	General laboratory	^9^
PCR	Food microorganisms such as *Escherichia coli*, *Salmonella*, *Helicobacter pylori*, *Staphylococcus aureus* etc.	10^2^ CFU/µL	Within 20 min	During the early onset of the infection, molecular biology, pathogen detection, medical diagnosis, food detection	Not suitable for large‐scale diagnostics, too many analytical steps, only one target can be monitored in one experiment	Medium cost, requires thermal cycler	Professional staff required	^208^
qPCR	*16S rRNA* gene COVID‐19	10^2^ ng/µL	30–120 min	Detection of *Lactobacillus helveticus* and *S. thermophilus* in all natural whey fermenter samples	Insufficiently sensitive and accurate for small nucleic acid concentrations	$10–20, fluorescent probe required	High clinical applicability, quantitative analysis	^113^, ^209^
RT‐PCR assay	Viruses RNA	10 copies/µL	≤25.5 min	Measurement of viral load during early episodes of infection	Requires trained personnel and specialised laboratories, expensive reagents	High cost, specialised reagents	High applicability, e.g. COVID‐19 detection	^208^
Digital PCR (dPCR)	DNA	3.8 copies/10^5^ cells	2–4 h	Genetically modified organism detection, molecular pathology and haematology for oncology, detecting circulating tumour DNA in cancer patients	Higher cost, lower flux capacity	Very high cost, microdroplet generation equipment	Precision medicine	^210^
LAMP	*Mycobacterium tuberculosis*	10¹ CFU/mL	≤35 min	Detection of *Mycobacterium tuberculosis*, *Mycobacterium avium* and *Mycobacterium intracellulare* in sputum specimens	Self‐priming or primer‐primer hybridisation produces false‐positive results, high risk of contamination, constant temperature required	Low cost, no complicated equipment required	Resource‐limited areas	^211^
RCA	Viruses RNA, foodborne microorganism	10^3^ copies/µL	Between a few hours and a day	Rapid food microbiological testing, miRNA analysis	Limited primer specificity, complicated design, amplification product length variation, and sensitivity to temperature result in longer processing times.	Medium cost	Research‐oriented	^212^
Conductivity biosensor measurements	Enterohemorrhagic *E. coli* O157:H7 and *Salmonella* spp, the *S* and *ORF1ab gene* sequences of SARS‐CoV‐2	100 CFU/mL	≤10 min	Detection of enterohemorrhagic *E. coli* O157:H7 and *Salmonella* spp, detect both the *S* and *ORF1ab* gene sequences of SARS‐CoV‐2, detecting circulating tumour DNA in cancer patients	Complex design and use, cross‐talk with other biomolecules, ions or chemicals	Medium cost, difficult sensor development	Laboratory research	^213^
IDA‐based IM sensors	*S. typhimurium*	4.8 CFU/mL	Between minutes and hours	Bacterial monitoring in complex media, biofilm analysis	Complexity, limited by detection limits, high cost of consumables, and long detection times.	High cost, sophisticated equipment	For industry and research	^9^
Next‐generation sequencing (NGS)	Viruses DNA, viruses RNA		1–7 days	Identification of unknown pathogens	Complex and time‐consuming data analysis, possibility of false positives.	High cost, specialised equipment	Complex disease analysis, unknown pathogen screening	^214^
Microfluidic technology	Viruses DNA, viruses RNA	10–100 copies/µL	30 min–2 h	On‐site testing in resource‐limited areas, micro‐sample analysis	High cost, reliance on precision manipulation, need to optimise pre‐processing steps	Low cost, specialised equipment	Immediate detection, multiple rapid screening and micro‐sample analysis	^215^

#### 3.1.2 Analysis of detection timeliness

The advent of CRISPR technology has engendered a substantial enhancement in the realm of assay timeliness, attributable to its fully process‐integrated design and enzymatic cascade reaction mechanism.^107^ Conventional isothermal amplification in combination with Cas protein detection is reported to require between 30 min and 1 h, while the microfluidic chip system has been shown to reduce this time to less than 30 min.^93^ This rapidity renders it suitable for immediate diagnosis at the bedside, such as postoperative infection testing,^108^ and aligns with the ‘precision medicine’ concept by enabling timely intervention, promoting its translation from theory to practice (early detection‐early typing‐early intervention) in prevention and control.^108^ In contrast, qPCR does not meet the requirements of emergency medicine because it requires thermal cycling for amplification, which takes between 60 and 90 min, resulting in a total time of 2–4 h.^109^ Culture methods are only suitable for the surveillance of chronic infections, due to the necessity of a 24‐h culture and 12–24 h of biochemical identification.^110^ The utilisation of ELISA technology in serological testing engenders a marked efficiency advantage, with a reaction time of 1–2 h for antigen‐antibody binding. This is in contrast to the 60‐min reaction time of conventional methods, which renders them ill‐suited to the screening of bulk samples.^111^

### 3.2 Cost and implementation feasibility assessment

#### 3.2.1 Economic comparison

The core advantage of CRISPR technology, with a single test cost of $2–5, lies in the portability of the equipment, including the microfluidic chip system^93^ and the test strip assay device,^112^ which significantly reduces the infrastructure investment. qPCR, with a single test cost of $10–20, is mainly derived from the average annual maintenance fee of thermal cycler of $5000–10 000 and fluorescence detection module^113^ accounting for more than 75% of the hardware cost. While the enzyme labelling instrument is relatively inexpensive, costing only $5–10 per run, the configuration of the ELISA system necessitates the acquisition of a costly enzyme labelling instrument^114^ that is priced at more than $5000. Furthermore, the deployment of the ELISA system in primary care settings is rendered more complex due to the requirement of additional large equipment, such as a plate washer, which increases the overall cost of implementation by three to five times. This limitation in terms of cost‐effectiveness restricts the applicability of the ELISA system in environments where resources are limited.^115^ The high cost of NGS is primarily attributable to the maintenance of sequencers ($5000–10 000 per year) and the reagents required for library preparation,^106^ while CRISPR technology has the potential to reduce infrastructure investment by 90% through the use of test strips or handheld devices.^112^ Despite the higher cost per sample of microfluidic chips in comparison to CRISPR, the unit cost of bulk samples can be reduced by parallel detection of multiple channels (e.g. 16‐channel chips).^116^ Microfluidic chips can be integrated into handheld devices,^117^ facilitating on‐site testing. Notably, test strip devices do not necessitate external power sources, a feature that renders them indispensable in scenarios such as infectious disease screening in remote regions, including malaria‐endemic zones and self‐testing in domestic settings.^118^ Conversely, qPCR and ELISA are contingent on thermostatic laboratories and professional operators with average annual electricity costs exceeding $2000, thereby ensnaring them within the confines of central laboratory systems.^119^ The disparities in technology economics are expediting a fundamental transition in the allocation of diagnostic resources.^120^ CRISPR technology is facilitating ‘clinic‐level testing’ in community pharmacies and even in domestic settings, while conventional technologies persist in their dominance within tertiary hospital testing facilities, which necessitate meticulous examination.^121^

#### 3.2.2 Progress on regulatory approvals

The prevailing diagnostic technologies currently present a regulatory landscape in which traditional methods and CRISPR systems are found to be analogous. Traditional PCR and ELISA have been widely approved by the FDA^122^ on the basis of mature clinical validation and their standardised processes, such as qPCR with a sensitivity of 1 copy/µL and ELISA with a specificity of >95%,^123^ are still the gold standard for laboratory diagnosis.

Recent years have seen significant regulatory breakthroughs in the field of CRISPR technology. For instance, the Cas13‐based SHERLOCK system has been granted Emergency Use Authorization (EUA) in the United States for the detection of new coronaviruses,^124^ while the Cas12a‐driven DETECTR technology has been CE‐certified for HPV typing with a specificity of 99.3%, marking the official entry of CRISPR diagnostics into clinical applications.^125^ The two technologies complement each other in terms of performance. The CRISPR system is able to meet the immediate diagnostic needs of grassroots level users due to its rapid 30 min detection and constant temperature operation.^126^ In contrast, the traditional technology maintains its position of high‐precision detection with automated equipment.

### 3.3 Clinical validation of empirical studies

#### 3.3.1 Pathogen detection efficacy

Innovations in molecular diagnostics are injecting new momentum into the prevention and control of infectious diseases. The utilisation of CRISPR‐SERS conjugation technology in the context of *M. tuberculosis* entails the integration of two distinct methodologies: the precise targeting of CRISPR‐Cas12a and the ultrasensitive detection of surface‐enhanced Raman spectroscopy.^127^ This integration reduces the detection limit to 4. The 42 pM, exhibiting a 10–100‐fold higher efficiency compared to qPCR.

It has been demonstrated that this assay achieves 100% accuracy in sputum samples and is capable of reducing the identification of multidrug‐resistant tuberculosis from the traditional ‘weekly’ level to the ‘hourly’ level.^49^ Furthermore, it has been shown to exhibit 98% concordance with resistance gene detection results with culture.^128^

For SARS‐CoV‐2 detection, the RT‐RAA‐Cas13a system complements the scenario with amplification‐free technology: the former achieves a portable detection of 1 copy/µL in 30 min through recombinant enzyme‐mediated amplification with the RNA shear property of Cas13a^124^; the latter circumvents false positives via double‐stranded displacement probes and cascade signal amplification, achieving a sensitivity of 0.47 copies/µL,^129^ and accurately capturing trace amounts of viruses in asymptomatic infected people and environmental samples.^130^

It is anticipated that, in the next 3–5 years, the enhancement of the WHO certification system will result in a transformation of the global infectious disease prevention and control paradigm.^131^ From the instant reading of the tuberculosis drug resistance gene to the ultra‐sensitive capture of the new coronavirus, from the on‐site typing of avian influenza subtypes to the integrated output of intelligent diagnosis and treatment, molecular diagnostics is breaking through the limitations of time, space and cost. This advances ‘precision medicine’ from theory (early detection‐early typing‐early intervention) to practical prevention and control, supporting efforts to end major infectious disease threats.^132^, ^133^

#### 3.3.2 Drug‐resistant bacteria detection applications

In the domain of clinical diagnostics, CRISPR‐Cas12a has been demonstrated to achieve simultaneous detection of 14 targets (attaining 100% concordance with PCR) of high‐risk HPV subtypes (16/18/31/33, etc.) through the utilisation of multiplexed gRNA array design and signal decoupling technology.^134^ Its magnetic bead enrichment strategy exhibits direct compatibility with cervical exfoliated cell samples, and the nucleic acid extraction‐free process results in a detection time of 1.5 h.^135^ This innovation addresses the issue of multi‐test interference while enabling low‐cost cervical cancer screening. The dCLISA assay, utilising a dual‐enhanced mechanism, achieves a 12‐fold sensitivity boost with 45 min pathogen/biomarker profiling, thus revolutionising infection control from retrospective analysis to real‐time intraoperative intervention.^46^

The capacity of CRISPR‐Cas12a to function across diverse domains is especially evident in the context of food safety monitoring.^60^ The development of an *invA* gene probe system for the detection of *Salmonella* combines the principles of culture‐free lysis and lyophilisation to achieve an ultra‐sensitive detection of 38 CFU/mL, thus achieving a 100‐fold increase in detection speed compared to conventional culture methods.^136^ The integration of smartphone AI readout technology with this system has been shown to enhance the efficiency of on‐site screening of fresh products by a factor of 10.^137^ This technological framework has also been demonstrated to have significant benefits for critical care medicine. The RPA‐CRISPR conjugation solution has been shown to achieve 30 min drug‐resistant phenotyping of ICU blood specimens (with 97% accuracy) through dual‐targeting assays with 16S rRNA internal reference quality control, thus transforming carbapenem dosing decisions from empirical 48‐h wait to real‐time precision intervention.^138^ The Cas12a system has been demonstrated to have the capacity to transform the current paradigm of infectious disease prevention and control, offering significant advantages in terms of multi‐target, ultra‐sensitive and intelligent functionality.^139^

### 3.4 Breakthroughs in cutting‐edge technologies

#### 3.4.1 No amplification detection

The development of a dual‐strand displacement probe with a hybridisation chain reaction signal amplification system enabled researchers to achieve amplification‐free direct detection of SARS‐CoV‐2 RNA with a sensitivity of 0.47 copies/µL.^129^ The technology converts the viral RNA binding event into a cascade of DNA self‐assembly reactions through a probe conformation switching mechanism, generating a detection signal with a 300‐fold increase in fluorescence intensity within 15 min.^140^ This reduces the risk of false positives by more than 40% compared to traditional RT‐qPCR (reverse transcription‐quantitative PCR), which relies on reverse transcription and thermal cycling amplification. This breakthrough design facilitates the immediate detection of low viral load samples, such as saliva and nasal swabs, which renders it particularly well suited for early screening of asymptomatic infected patients and vaccine efficacy assessment scenarios.^141^

#### 3.4.2 Portable device integration

A smartphone‐integrated CRISPR detection system combines optical signal analysis (1 copy/µL sensitivity) with blockchain encryption to ensure secure data flow. Detection results are time‐stamped, encrypted via elliptic curve algorithms and stored on tamper‐proof blockchain nodes^93^ through smart contract verification.^93^, ^142^ This architecture enables judicial‐grade data integrity for Ebola monitoring and antibiotic resistance tracking, eliminating risks of paper record loss or electronic report tampering.^142^

#### 3.4.3 Multiple testing platforms

The microfluidic chip system developed utilising CRISPR‐Cas12a in conjunction with multiplexed nucleic acid amplification technology has achieved simultaneous identification of eight pathogens, including influenza A/B viruses,^143^ respiratory syncytial virus (RSV) and SARS‐CoV‐2. The integration of a 16‐channel microreaction chamber with a fluorescence signal spectrometry system (sensitivity up to 10 copies/µL) has yielded results concordant with those of qPCR at a rate of 99.2%.^144^ The chip utilises centrifugal force‐driven sample distribution technology, thereby compressing the entire nucleic acid extraction, isothermal amplification and targeted detection process into a mere 45 min. The chip has successfully completed the technical validation stage for CE marking, and it is anticipated that it will become a rapid typing tool for respiratory infections in emergency departments and community clinics upon approval at the end of 2024. It is estimated that the chip will reduce the risk of cross‐contamination by 80% in comparison with traditional testing systems.

#### 3.4.4 Gene editing optimisation

The engineered FnCas9 variant was subjected to reconstruction of the α‐helical structural domain and optimisation of the PAM recognition interface, which resulted in a significant 3.5‐fold enhancement in single‐base mismatch tolerance.^145^ Subsequent mechanistic elucidation demonstrated that the mutant K538A/R544Q two‐site modification enhanced the topology of the gRNA‐DNA heteroduplexes, thereby achieving a single‐nucleotide polymorphism differentiation accuracy of 99.7%.^146^

This precise identification technology has been successfully translated into clinical‐grade testing tools.^147^ As demonstrated in the relevant literature, these tools have been shown to achieve specific differentiation of 0.1% mutation abundance in lung cancer.^121^ Moreover, these findings have the potential to provide a new generation of molecular probe development platforms for tumour liquid biopsy and rapid testing of tuberculosis drug‐resistant genes.^148^ The CRISPR diagnostic system has been demonstrated to demonstrate superiority over conventional methods in terms of three‐dimensional advantages of sensitivity, speed and cost‐effectiveness.^149^ The advancement of the aforementioned technology towards CE/FDA certification, coupled with its 97% clinically validated accuracy, provides a robust empirical foundation for the utilisation of precision medicine applications.^150^

## 4 THE INNOVATIVE DIRECTION OF THE NEXT‐GENERATION CRISPR DETECTION TECHNOLOGY

### 4.1 Multiplex detection and high‐throughput platform

The evolution of CRISPR‐enabled bacterial detection systems is focused on overcoming practical implementation barriers while achieving quantifiable reliability metrics,^8^, ^151^ thereby meeting stringent requirements for point‐of‐care applications in infectious disease management, pathogen screening and ecosystem microbial surveillance. In this context, the multiplex CRISPR detection system demonstrates remarkable performance characterised by ultra‐high sensitivity, exceptional specificity and high‐throughput parallel detection capacity. The multiplex CRISPR detection system enables multiplex assays within the same experimental system or reaction conditions, allowing for the simultaneous identification of multiple target molecules in a single experiment (such as pathogen nucleic acid, protein markers, cell signalling molecules etc.), thereby breaking through the limitation of traditional single weight detection and providing robust technical support for real‐time monitoring applications. For instance, integrated CRISPR‐Cas systems with nanosensors, nanopore sequencing and AI‐assisted technologies enabled ultrasensitive pathogen detection via pre‐amplification‐enhanced multiplexed assays (fluorescence/electrochemical/lateral flow^152^), enhanced single‐cell gene editing efficiency and targeted enrichment strategies.^153^ This platform achieved single‐molecule pathogen detection,^152^ deep *AMR* gene analysis (30‐fold improved detection depth), and rapid clinical sample screening (2‐h turnaround time, AUC = 1^154^). It provides a high‐throughput, low‐resource adaptable multiplexed biosensing platform for point‐of‐care diagnostics of foodborne pathogens, genetic disorders and cancers, while accelerating gene therapy drug development.

With the breakthrough of microfluidic technology and digital CRISPR system, multiple detection is developing towards higher throughput and automation. For instance, the integration of microfluidic technology with CRISPR systems enables multiplex detection of five or more targets, achieving results in just 30 min.^155^ Furthermore, this technology demonstrates exceptional performance in automated clinical sample processing, with both sensitivity and specificity reaching 100%.^156^ It not only enhances the throughput of pathogen detection but also significantly improves detection sensitivity and automation capabilities, providing robust support for the advancement of telemedicine and infectious disease surveillance.^157^ This technological breakthrough enables the miniaturisation of traditional detection requiring large equipment (such as digital PCR), and at the same time, through the specific recognition ability of CRISPR, a new paradigm of ‘miniaturisation and high precision’ detection is constructed. It is expected to replace traditional fluorescent qPCR.^158^

### 4.2 Amplification‐free CRISPR detection: Towards simplified diagnostics

In recent years, non‐amplified CRISPR detection technology has made significant breakthroughs in sensitivity, timeliness and clinical applicability through the collaborative innovation of multiple strategies. Taking the CRISPR‐Cascade system as an example, it achieves rapid diagnosis of bloodstream infections through precise control of cascade reactions, completing tests within 10 min with a plasma sample compliance rate of 98.7%.^159^ Compared to traditional PCR, this technology does not require thermal cycling steps, fundamentally avoiding the risk of false positives caused by aerosol contamination during nucleic acid extraction. In terms of optimising the detection system, researchers have successfully eliminated the dependence on pre‐amplification steps by reconstructing the spatial conformation of crRNA using computer‐aided design, while reducing the probability of cross‐contamination to below 0.1%.^160^ The breakthrough in signal amplification strategies is even more remarkable: The CrisprAIE system innovatively couples Cas12a protein with aggregation‐induced‐luminescence probes, utilising the fluorescence enhancement effect triggered by nanoparticle aggregation to achieve sensitive detection of *norovirus/*SARS‐CoV‐2 (up to 80–270 times) without nucleic acid amplification. This system further integrates smartphone imaging modules, enabling high‐precision data analysis in remote area laboratories using common equipment.^161^ In response to the complexity of clinical samples, PMA pre‐treatment combined with digital CRISPR/Cas12a technology demonstrates unique advantages: selective labelling of live bacterial nucleic acids with photosensitive dyes reduces the detection limit for live *S. aureus* O157:H7 to 1.2 × 10^3^ CFU/mL, achieving a quantitative correlation *r* = 0.949 with plate culture methods.^162^ The parallel development of non‐CRISPR systems also deserves attention: The Argonaute protein (TtAgo/PfAgo) achieves aM‐level pathogen detection without amplification, with a single‐base mismatch resolution rate of 99.3%.^163^ The synergistic system of DNAzyme and CRISPR‐Cas12a expands the detection dimension – this platform not only identifies environmental pollutants such as lead ions (Pb^2^⁺) but also targets prostate cancer by cleaving oncogenic mRNA, achieving a detection limit as low as 5 aM.^164^ In the field of viral detection, the Cas13a system establishes a rapid diagnostic process without RNA extraction by directly recognising *IAV* segment 5 mvRNA fragments in nasopharyngeal swabs, securing a golden window for influenza antiviral treatment.^165^ These advances collectively establish non‐amplified CRISPR‐based detection as a transformative approach, combining ultrahigh sensitivity, rapid turnaround and robust clinical adaptability without thermal cycling dependencies.

### 4.3 Intelligent CRISPR systems

The combination of artificial intelligence and CRISPR is reshaping the paradigm of diagnosis, CRISPR signal can be used with smart phones (the results are digitised and transmitted remotely), so that real‐time data analysis can be carried out. For instance, CrisprAIE system (Cas12a + aggregation induced luminescence probe Q‐dsDNA/AIEgens‐Q) can detect *norovirus/*SARS‐CoV‐2 without amplification; the sensitivity is increased by 80–270 times, and the sensitivity is further improved after SNA/AIEgen modification, suitable for smartphone reading.^161^ In addition, the combination of Cas9/Cas12/Cas13h and nanopore sequencing for multiple detection (such as cancer gene mutation + pathogen) can be enhanced with AI to improve its sensitivity and promote the application of POCT in infectious diseases (tuberculosis, malaria) and genetic diseases.^166^ This synergy not only enhances detection sensitivity but also paves the way for next‐generation POCT devices combining CRISPR's specificity with AI‐driven data analysis.

## 5 CLINICAL APPLICATIONS AND TRANSLATIONAL CHALLENGES

### 5.1 Technical challenges

#### 5.1.1 Inhibitor interference in complex sample pre‐processing

The presence of inhibitors (such as mucins and haemoglobin) in clinical samples (like sputum and blood) significantly hampers the sensitivity and reliability of CRISPR‐based detection methods. Studies have demonstrated that mucins in sputum can reduce the activity of Cas12a by 50%,^167^ while haemoglobin in blood inhibits Cas enzyme functionality through oxidative reactions.^168^

To address these challenges, numerous contemporary studies have proposed innovative solutions to facilitate clinical translation: for instance, as previously mentioned, the DETECTR technology successfully enabled direct detection of HPV DNA in unprocessed clinical samples without nucleic acid extraction. This method exhibits remarkable tolerance to PCR inhibitors, significantly streamlining the sample pre‐treatment workflow by eliminating time‐consuming purification steps that may introduce sample loss or experimental variability.^3^ At the same time, using target enrichment techniques to enable magnetic nanoparticles to selectively adsorb target nucleic acids, combined with surface modifications (such as polyethyleneimine) to enhance interference resistance, which can elevate the detection limit of sputum samples to 10 copies/µL.^169^ Additionally, by integrating sample lysis with RPA amplification in a ‘one‐pot’ method, we can directly detect *K. pneumoniae* in whole blood samples. This non‐extraction strategy is expected to reduce pre‐processing steps.^168^ Furthermore, by leveraging the auxiliary functions of specific enzymes, we introduce a heat‐stable DNA polymerase that synergises with Cas12a for target amplification in sputum without the need for purification, achieving sensitivity at the aM level,^170^ thus optimising amplification techniques. Recent studies show that a CRISPR‐SERS method can detect *M. tuberculosis* at concentrations as low as 4.42 pM 10–100 times more sensitive than qPCR. It achieved 100% accuracy in sputum samples and reduced detection time for drug‐resistant *M. tuberculosis* from weeks to hours.^49^ The technique also showed 98% agreement with culture results for drug resistance genes, demonstrating strong potential for use with complex samples.^128^

#### 5.1.2 Scalability bottlenecks in high‐throughput detection

Current CRISPR platforms struggle to meet the demands of large‐scale clinical screening due to limitations in throughput (typically <50 samples per batch) and automation levels. Although microfluidic technology has significantly enhanced detection efficiency, it has also increased equipment costs by 30%.^156^ In response, we aim to gradually overcome these limitations through innovations in digital intelligence technology: firstly, by integrating digital microfluidics, where microfluidic chips based on electrowetting effects can achieve parallel detection of 96 samples/h. When combined with the CRISPR/Cas12a system, the accuracy of *Helicobacter pylori* typing is expected to exceed 98%.^156^ A deep learning‐driven microfluidic platform^171^ automates the analysis of CRISPR fluorescence signals through image recognition, with this intelligent system development poised to increase throughput to 200 samples/h while also reducing manual interpretation errors. The label‐free detection technology represents a significant advancement in clinical applications, wherein a fibre‐optic‐coupled CRISPR‐based sensor^172^ utilising the evanescent wave principle enables direct detection of *E. coli* O157:H7 without requiring nucleic acid amplification steps, making it particularly suitable for on‐site rapid screening.

### 5.2 Implement obstacle analysis

#### 5.2.1 Demand for multi‐centre clinical data

The clinical translation of CRISPR diagnostic technology (CRISPR‐Dx) requires rigorous validation through multi‐centre trials. A CRISPR/Cas‐based diagnostic platform for high‐risk HPV exhibits 100% concordance with PCR while simultaneously detecting 14 types and is compatible with cervical exfoliated cell samples, delivering results within 1.5 h.^125^ On the other hand, the study by Wang et al. only used 40 pork and environmental samples from 3 retail markets for practical testing, with a small sample size and limited geographical scope. Although the detection system showed high sensitivity and specificity in these samples, the data were still insufficient to represent broader real‐world application scenarios.^173^ Additionally, the absence of standardised quality control has exacerbated the complexity of technology implementation. Through high‐throughput screening on the Echo liquid handling platform and optimisation of reaction parameters using the ODE mathematical model (e.g. the observed negative correlation between T7 RNAP concentration and detection signal),^170^ minor variations in enzyme concentration, buffer composition and other factors were found to cause fluctuations in results. This highlights that parameters optimised in a single laboratory may not be directly transferable to other research sites. Therefore, establishing unified operational protocols through multi‐centre data is essential, including defined thresholds for enzyme concentrations, standardised primer design criteria and other critical variables.^174^

#### 5.2.2 Regional regulatory differences

There are significant discrepancies in the evaluation standards for CRISPR‐Dx between the FDA and EMA. The FDA mandates a detection specificity of >99.5%, while the EMA requires only 98%.^51^, ^125^ This lack of standardisation compels companies to adjust their validation strategies for different markets, thereby increasing research and development costs and timelines.

#### 5.2.3 Cost competitiveness model

Although the cost of a single CRISPR test can be as low as $2–5,^62^ the financial obstacles to its large‐scale application still deeply root in the structural characteristics of the medical system. Take the portable microfluidic system as an example, its initial investment amounts to $500–2000 per unit.^112^ In the long term, CRISPR could save an average of $50 000 annually in reagent costs.^50^ Its advantages are even more pronounced in resource‐limited areas, where total costs are 80% lower than traditional culture methods.^62^ More revolutionary is the breakthrough in inkjet‐printed biosensors‐through the direct‐writing technology of nano‐silver conductive ink, the manufacturing cost of CRISPR electrochemical sensors has been reduced to $1.5 per test.^175^

#### 5.2.4 Lack of standardisation

The sensitivity fluctuations of commercial kits (such as a 20% variance in Cas14 reagents) highlight issues like non‐transparent buffer formulations.^13^ In multiplex detection, the lack of standardised crRNA concentrations has led to poor comparability of data between laboratories. To this end, the mandatory implementation of the ‘MIQE‐CRISPR’ guidelines should not stop at concentration standardisation but rather establish a full lifecycle traceability system for molecular diagnostics. A pilot project by the German Cancer Research Center has confirmed that by analysing the conformational stability of the Cas14‐buffer complex via cryo‐electron microscopy and combining machine learning to predict the optimal ion combination, the batch‐to‐batch variation of reagent kits can be reduced to within 4%.^170^, ^176^

### 5.3 Translational medicine strategy

#### 5.3.1 Collaborative innovation between industry, academia and research

The clinical translation of CRISPR‐Dx technology relies on interdisciplinary collaboration and industrial chain integration. A one‐tube RPA‐CRISPR‐Cas12b‐based detection system enabled the specific detection of mcr‐1 and tet (X4). The transition from laboratory validation to pre‐commercial production was accomplished within 18 months, with the core effort being the joint optimisation of crRNA design and reaction systems by universities and companies.^173^ A microfluidic‐CRISPR platform has been developed for the multiplex detection of eight respiratory pathogens. The assay shows 99.2%^144^ agreement with qPCR and is expected to obtain CE marking by late 2024, facilitating its adoption as a point‐of‐care diagnostic tool in urgent care and primary practice settings. Additionally, the digital RPA‐CRISPR platform,^177^ which combines microfluidic chips with cloud computing, has achieved precise quantification of hepatitis B virus DNA and is currently in the multi‐centre validation stage.

#### 5.3.2 Policy drivers and optimisation of regulatory frameworks

Global regulatory agencies are promoting the implementation of CRISPR‐Dx through accelerated approval pathways. The FDA's ‘Breakthrough Device Program’ prioritises the review of HPV genotyping kits and allows the sharing of multi‐centre trial data to reduce redundant validation.^13^ The European Union, through the ‘CRISPR‐Dx Acceleration Program,’ coordinates EMA and member state standards, explicitly requiring a sensitivity threshold of ≤1 aM and including cross‐population verification requirements for resistance gene testing.^178^ Furthermore, the International Organization for Standardization (ISO) is drafting performance validation guidelines for CRISPR diagnostic devices, which will cover parameters like clinical sample types, inhibitor tolerance thresholds and batch‐to‐batch consistency.^170^

#### 5.3.3 Technology dissemination and empowerment at the grassroots level

In resource‐poor areas, low‐cost technology adaptation and localised training are key. Inkjet‐printed CRISPR biosensors^62^ have reduced the cost of individual tests to $1.50 and can be powered by a solar module for use in off‐grid environments. The ‘CRISPR Community Health Project’ in Africa covers 500 healthcare institutions, and through standardised operational videos and remote quality control systems, it has reduced the error rate from 30% to 8%.^179^

### 5.4 Ethics and biosafety

The widespread application of CRISPR technology in pathogen detection and monitoring has improved diagnostic efficiency but has also raised significant ethical and biosafety concerns. In wastewater monitoring^180^ and wildlife pathogen testing,^181^ CRISPR tools may enter the natural environment through sample residues or equipment leaks. If CRISPR sensors used in wastewater are not fully inactivated, their Cas proteins or crRNAs could be assimilated by environmental microorganisms, unintentionally editing non‐target genes. Research indicates that CRISPR components can have a half‐life of up to 72 h in natural water bodies, increasing the risk of lateral gene transfer. Additionally, portable detection devices often lack standardised safety designs, leading to the potential spread of inactivated CRISPR components through soil or water post‐use.^181^

Genetic barcodes associated with CRISPR (such as specific spacer sequences) could also be captured by environmental microorganisms, altering their evolutionary paths. For instance, artificially designed CRISPR arrays might be integrated by bacteriophages, resulting in unpredictable cascading effects within ecosystems. Furthermore, biosafety regulations for the global field application of CRISPR are still inadequate, posing risks of misuse, such as the reversal of crRNA designs from test kits for bioweapons development.^182^

To mitigate the biosafety risks associated with CRISPR technology, the scientific community has proposed a multifaceted array of countermeasures in recent years. Technologically, Ye et al. developed self‐deactivating CRISPR systems using engineered Cas proteins (e.g. pH‐/temperature‐sensitive variants). Their design incorporates a TEV protease cleavage site in Cas9, enabling automated degradation after detection.^183^ Complementing these biological safeguards, physical containment methods like PDMS‐gelatin microfluidic encapsulation provide additional protection by maintaining CRISPR reagents in a thermostable state until high‐temperature activation (>60°C), effectively preventing environmental leakage.^28^ Additionally, we recommend establishing a global biosafety database, mandatory biosafety certification for devices (BSL‐2 and above),^184^ and inclusion in the revision framework of the Cartagena Protocol. Furthermore, we suggest implementing a review mechanism for open‐source sharing of CRISPR technology, with restrictions on the cross‐border circulation of high‐sensitivity detection tools. By adopting the aforementioned integrated strategy that combines technology, regulation and ethics, we aim to ensure the sustainable development of CRISPR applications in public health.

### 5.5 Future direction

#### 5.5.1 Integrated ‘sample in‐result out’ system

Next‐generation CRISPR diagnostic devices will integrate the entire process of nucleic acid extraction, amplification and detection. By adding fully automated nucleic acid magnetic bead‐extracted samples to a pre‐packaged centrifugal microfluidics chip, 48 samples can be tested automatically within 15–60 min at 60°C.^185^ To enhance accessibility, a smartphone‐based capillary sensor integrates CRISPR/Cas13a detection with artificial intelligence to analyse fluorescence images, enabling rapid and equipment‐free quantification of nucleic acids. This system supports real‐time cloud‐based data reporting, facilitating remote diagnostics and epidemiological surveillance in point‐of‐care settings.^37^, ^186^ Such innovations are critical for point‐of‐care, home‐based and resource‐limited settings, but challenges remain in cost reduction, shelf stability and minimising user‐dependent variability.

#### 5.5.2 Powering global validation with integrated smart and sustainable technologies

Standardised validation across diverse populations is essential to address geographical biases in pathogen variants and host genetic backgrounds. Future initiatives must prioritise inclusion of low‐income regions and establish open‐access databases (e.g. CRISPR‐Dx Global Hub) to share detection performance metrics, inhibitor interference profiles (e.g. heparin, haemoglobin) and resistant mutation hotspots impacting guide RNA binding.^187^

AI‐driven CRISPR design platforms are revolutionising assay development. CRISPR‐GPT utilises a multi‐agent large language model framework, fine‐tuned on domain‐specific data, to automate the design of gene‐editing experiments. This system reduces the experimental planning workflow from several weeks to mere minutes while maintaining high accuracy and achieving editing efficiencies exceeding 80% in validation studies.^188^ In a complementary approach, OpenCRISPR‐1 leverages AI‐driven protein language models trained on a curated atlas of over one million CRISPR operons to generate novel Cas effectors. The resulting editor achieves on‐target efficiency comparable to SpCas9, reduces off‐target effects by 95%, and exhibits significantly lower immunogenicity, thereby enhancing its potential therapeutic compatibility.^189^Moreover, the combination of CRISPR and synthetic biology^168^ enables the design of ‘detection‐treatment’ integrated systems. For example, CRISPR‐Cas12a circuits can be engineered to trigger the in situ synthesis of antimicrobial peptides upon pathogen detection, as demonstrated in recent studies.^190^

## 6 CONCLUSION

The CRISPR/Cas system has emerged as a transformative tool in the field of molecular diagnosis, offering unprecedented sensitivity, specificity and rapid detection capabilities. Through the dual mechanisms of programmable nucleic acid recognition and enzyme‐mediated signal amplification, CRISPR‐based assays surpass conventional PCR methods in workflow simplicity and cost‐effectiveness ($2–5 per test). Clinical validations demonstrate practical utility in detecting drug‐resistant bacteria and emerging viruses. Moreover, multiplex platforms enable simultaneous analysis of up to 14 targets, significantly expanding its application potential in complex sample detection. However, several challenges must be addressed in clinical translation, including sample matrix interference, standardisation gaps and regulatory disparities between regions. In the future, the convergence of CRISPR with synthetic biology, nanotechnology and artificial intelligence promises to attach fully automated ‘sample‐in‐result‐out’ systems and genome‐scale pathogen surveillance networks. Such advancements will not merely transform infectious disease management but empower decentralised healthcare in resource‐limited settings, marking a new era of precision diagnostics and global health equity.

## AUTHOR CONTRIBUTIONS

All authors took part in writing, reviewing and editing the manuscript. Zilong Wang, Bingyu Li and Yuke Li wrote the manuscript; Qianqian Wang and Dandan Guo prepared the figures and the tables; Zhengbo Chen and Jiaming Zhang collected and organised the literature; Shuying Feng modified the paper. All authors reviewed the manuscript and approved it for publication.

## CONFLICT OF INTEREST STATEMENT

The authors declare no conflicts of interest.

## ETHICS STATEMENT

This article does not contain any studies with human participants performed by any of the authors.

## Data Availability

All data of this article are included within the article. It can also be requested from the corresponding or first author.
